# Long-term *In Vivo* Tracking of Inflammatory Cell Dynamics Within *Drosophila* Pupae

**DOI:** 10.3791/57871

**Published:** 2018-06-14

**Authors:** Helen Weavers, Anna Franz, Will Wood, Paul Martin

**Affiliations:** ^1^School of Biochemistry, Biomedical Sciences, University of Bristol; ^2^School of Cellular and Molecular Medicine, Biomedical Sciences, University of Bristol; ^3^MRC Centre for Inflammation Research, University of Edinburgh, Queens Medical Research Institute; ^4^School of Physiology, Pharmacology, and Neuroscience, Biomedical Sciences, University of Bristol

**Keywords:** Immunology and Infection, Issue 136, Wound healing, inflammation, innate immunity, *Drosophila melanogaster*, *Drosophila* pupa, hemocyte, cell migration, live-imaging, laser ablation, confocal microscopy, photoconversion

## Abstract

During the rapid inflammatory response to tissue damage, cells of the innate immune system are quickly recruited to the injury site. Once at the wound, innate immune cells perform a number of essential functions, such as fighting infection, clearing necrotic debris, and stimulating matrix deposition. In order to fully understand the diverse signaling events that regulate this immune response, it is crucial to observe the complex behaviors of (and interactions that occur between) multiple cell lineages *in vivo, *and in real-time, with the high spatio-temporal resolution. The optical translucency and the genetic tractability of *Drosophila *embryos have established *Drosophila *as an invaluable model to live-image and dissect fundamental aspects of inflammatory cell behavior, including mechanisms of developmental dispersal, clearance of apoptotic corpses and/or microbial pathogens, and recruitment to wounds. However, more recent work has now demonstrated that employing a much later stage in the *Drosophila *lifecycle – the *Drosophila *pupa – offers a number of distinct advantages, including improved RNAi efficiency, longer imaging periods, and significantly greater immune cell numbers. Here we describe a protocol for imaging wound repair and the associated inflammatory response at the high spatio-temporal resolution in live *Drosophila *pupae. To follow the dynamics of both re-epithelialization and inflammation, we use a number of specific *in vivo *fluorescent markers for both the epithelium and innate immune cells. We also demonstrate the effectiveness of photo-convertible fluorophores, such as Kaede, for following the specific immune cell subsets, to track their behavior as they migrate to, and resolve from, the injury site.

**Figure:**
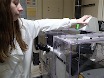


## Introduction

An efficient and effective inflammatory response is pivotal for any organism to fight infection, clear debris, and orchestrate the repair of injured tissues^1^. Although this response is an inevitable outcome of most tissue damage, inflammation requires strict regulation because an inappropriate inflammatory response has been linked to a variety of different human diseases (including chronic non-healing wounds, excessive scarring, and predisposition to cancer)^1^,^2^,^3^. Given this clinical relevance, it is crucial to obtain a more detailed understanding of the molecular and cellular mechanisms driving the inflammatory response in order to develop new prognostic indicators and strategies to treat a range of chronic inflammatory conditions, which might protect repairing tissues from prolonged and unnecessary inflammation.

In recent years, *Drosophila* has become a well-established and valuable model system to dissect fundamental features of the inflammatory response conserved from insects to human^4^,^5^. At present, *Drosophila *offers far greater genetic tractability than is currently possible in other experimental models (such as mice or zebrafish), permitting precise spatio-temporal genetic manipulation *in vivo* (to inactivate or over-express any gene of interest within specific cell types at a defined developmental time-point) and ease of genome-wide screens^6^,^7^. Traditionally, most live-imaging studies of wound healing and inflammation in *Drosophila *have been performed at embryonic stages, as embryos are immobile (unlike *Drosophila *larvae or adults) and optically translucent which enables unparalleled high resolution *in vivo* imaging^8^. This has allowed researchers to visualize the rapid and robust recruitment of *Drosophila *innate immune cells (hemocytes) to the wound site in response to the mechanical or laser-induced injury to the embryonic epithelium^9^,^10^,^11^,^12^,^13^,^14^. By combining these live-imaging studies with the genetic manipulation, studies in *Drosophila *embryos have uncovered many important immune cell proteins that control inflammatory cell behavior *in vivo*. For example, the CED-1 homolog Draper (an ITAM domain-containing protein) has been identified as an important 'damage receptor' that mediates the recruitment *Drosophila *immune cells in an H_2_O_2_-dependent manner to sites of damage^15^. Draper levels within immune cells are in turn regulated by calcium-induced JNK signaling and elevated downstream of apoptotic corpse uptake^12^. Hemocyte motility further requires complex cytoskeletal changes to coordinate directed migration towards the wound and this is dependent on the activity of cytoskeletal regulators such as the actin-bundling protein Fascin^16^ and the Rho family small GTPases Rac and Rho^9^.

*Drosophila *is a holometabolous insect that goes through the additional larval and pupal stages following embryogenesis, prior to reaching adulthood^17^. The *Drosophila* pupa has been developed as an additional model for non-invasive live-imaging of a variety of dynamic cellular events, including developmental cell migration^18^, cell division^19^, cell growth^20^, and muscle contraction^21^. More recently, it has been established as a novel model in which to study the dynamics of wound repair and inflammation *in vivo*^22^,^23^.

Just as in embryonic stages, *Drosophila *pupae are immobile and optically translucent, after careful dissection from their opaque pupal cases^18^. By exploiting this optical transparency, one can follow the *in vivo *behavior of innate immune cells (hemocytes) in response to the tissue damage within *Drosophila *pupal tissues, such as the pupal wing^22^. The pupal wing exists as a simple bilayered structure, consisting of two large flat epithelial sheets that are connected around the wing periphery; the extracellular space between these two epithelial layers is filled with hemolymph (insect blood) and large numbers of motile hemocytes^24^. Just as in embryos, mechanical or laser-induced injury to the wing epithelium triggers a rapid recruitment of hemocytes to the injury site^23^. However, this pupal stage offers some distinct advantages for imaging over earlier embryonic stages. Injured pupae can be imaged over far longer time periods (for at least 5 h), more tissue area is available for the experimental perturbation (such as generation of multiple wounds) and there are significantly greater numbers of hemocytes present at this stage (providing more cell trajectories from further distances for improved statistical power during the mathematical analysis). Furthermore, the efficiency of RNAi-mediated gene inactivation is considerably improved in pupal stages, allowing many genes to be 'knocked-down' in a tissue or the time-specific manner compared to the more traditional whole mutant approach of embryos.

In order to follow the dynamics of wound re-epithelialization and the accompanying inflammatory response within this new pupal model, two different cell populations must be labeled: the pupal epithelium and the innate immune cells (*Drosophila *hemocytes). A number of different markers (**Table of Materials**) are available to label these two different cell populations - the choice of marker depends on the particular process to be studied. To mark the pupal epithelium, *Drosophila *lines containing either a ubiquitously expressed GFP-tagged E-cadherin (that labels adherens junctions) are routinely utilized to indicate the positions of the cell margins, or alternatively, a GFP-tagged Actin-binding domain of Moesin (that labels the actin cytoskeleton) to visualize the wound-edge contractile actin ring and leading-edge protrusions. To label the *Drosophila *hemocytes, a hemocyte-specific *srp-Gal4*^25^ to drive expression of nuclear RFP (for nuclear tracking), cytoplasmic GFP or GFP-tagged Moesin (to label the cytoplasm or actin cytoskeleton, respectively) or a photoconvertible fluorophore (such as Kaede) are used. In fact, it is often advantageous to use multiple immune cell markers in combination, to enable simultaneous analysis of the nuclear movement and the cell morphology (see Representative Results). However, since this protocol involves the use of *Drosophila *pupae, only combinations of genetic markers that are viable until mid-pupal stages can be utilized. Also, embryonic lethal stocks will not be suitable. This is unlikely to be a problem when imaging control (or wild-type) pupae but is important to consider when genes are to be knocked down or overexpressed, to assess their effect on the wound closure or inflammation. In the case of early lethality caused by gene knockdown (or overexpression), a Gal80^ts^ construct can be used to induce the Gal4-driven knockdown later in development (see Discussion).

In our recent studies, moving into the pupal stage has enabled us to gather sufficient immune cell trajectory data to analyze inflammatory behavior using sophisticated mathematical modeling, which in turn has allowed us to deduce novel details of the wound inflammatory attractant signals^23^. For example, this approach revealed that the wound chemoattractant spreads slowly through the inflamed tissue at 200 μm^2^/min, a rate far slower than previously suggested small candidate molecules such as ATP or H_2_O_2_ are reported to diffuse^26^,^27^,^28^,^29^; these small "damage" molecules are instead likely to act as permissive signals. Moreover, by following the long-term behavior of innate immune cells as they resolve away from an initial wound and are exposed to a second (made at various timepoints after the first), we have uncovered a temporary 'desensitization' period during which immune cells are blind to subsequent injuries^23^. By exploiting the long-term imaging potential of the pupal model, together with *Drosophila's *genetic tractability, one can follow the behavior of specific immune cell populations (such as only those immune cells recruited to the wound site) in response to subsequent insults, using the photoconvertible fluorophore^30^ which can be expressed exclusively within the immune cell lineage^23^.

Here we describe a protocol to visualize the dynamics of wound repair and the associated inflammatory response at high spatio-temporal resolution using living *Drosophila *pupae. We provide a detailed methodology to cover the steps required for the initial pupae preparation (dissection and mounting) and the subsequent laser-mediated wounding and the time-lapse imaging. We also describe the use of photo-convertible fluorophores to permit the labeling of specific immune cell subsets *in vivo*. In the long-term, we envision that this new *Drosophila *pupal model will open up exciting possibilities for dissecting the complex signaling dynamics underlying the inflammatory response to tissue damage. By applying more sophisticated statistical analyses one might uncover features of the response that would otherwise remain experimentally inaccessible, whilst the improved RNAi efficiency could lend itself to the application of genome-wide screening within immune cells *in vivo *to identify novel players regulating immune cell behavior.

## Protocol

This protocol consists of four main sequential steps: (1) Preparation of *Drosophila *stocks and staging of *Drosophila *pupae, (2) Pupal dissection and mounting, (3) Pupal wounding, (4) *in vivo *time-lapse confocal imaging.

### 1. Preparation of *Drosophila *Stocks and Staging of Pupae

1. Obtain appropriate *Drosophila *stocks (see Introduction and **Table of Materials**).
2. Collect young healthy adult flies of the appropriate genotype.
  1. Select adult flies by using carbon dioxide gas pads to briefly anesthetize the flies and a fine paintbrush to transfer flies of the appropriate genotype or gender to a collection vial.
  2. Add 20 virgin females and 20 males to each vial containing standard fly food media (a cornmeal-molasses-agar mixture, see **Table of Materials**) supplemented with yeast.
3. For the optimal pupal generation, tip the adult flies each day onto new food in fresh vials, keeping all vials at 25 °C. Note: If the Gal4-UAS system^31^ is being used to drive lineage-specific gene expression, all steps should be performed at 25 °C or above, as the Gal4-UAS system is temperature sensitive.
4. 18 h before the scheduled imaging session, select at least 10 newly formed white pre-pupae (Figure 1A) from the vials (*i.e.* 0 h after puparium formation, APF) using forceps or a fine paintbrush to dislodge pupae from the interior vial surface and transfer pupae carefully to the side of a clean empty plastic vial. Note: Wandering 3^rd^ instar larvae crawl upwards out of the food medium to undergo pupation; newly formed white prepupae are easily identified as they possess everted anterior spiracles and are stationary (unlike 3^rd^ instar larvae). The cuticle transforms into the ‘puparium’ (the pupal case) which is initially soft and white^17^. Care must be taken to avoid damaging the pupae, as this can lead not only to an unwanted injury-induced inflammatory response but also to a significant developmental delay.
5. Age the selected pupae to the appropriate developmental stage (18 h APF will give optimal results) in the vial at 25 °C. Note: As the pupae develop, the pupal case will become progressively darker and more brittle.
6. Prepare other reagents for the next step ahead of time. To make the heptane glue, combine a 20 cm length of rolled up double-sided tape with 20 mL heptane in a 50 mL centrifuge tube, seal with the paraffin film and rock at the room temperature overnight on the bench.

### 2. Preparation and Dissection of *Drosophila *Pupae

1. Transfer the staged *Drosophila *pupae to a piece of double-sided sticky tape mounted on a glass slide. Position the pupae so that the ventral side is firmly stuck to the tape and the dorsal side is facing upwards (Figure 1B). Note: The anterior of the pupa will be identifiable from the two spiracles protruding from the anterior end of the pupal case.
2. Carefully remove pupae from their protective puparium casing under a brightfield dissection microscope using forceps and micro-scissors (Figure 1B**-D**).
  1. Initially, make an incision in the anterior-most region of the puparium using the forceps (Figure 1B). Ensure that the pupal case in this area is hollow and devoid of pupal tissue because the pupae will have shrunk within the case during early pupal development.
  2. After this initial incision, carefully tear or cut the pupal case open in an anterior to posterior direction using forceps or microscissors (Figure 1C) until the pupa is completely free from the brown opaque brittle casing (Figure 1D). Note: Pupae at this stage are very fragile and care must be taken to avoid puncturing the pupal surface; puncturing is obvious as hemolymph quickly leaks out from the puncture site. Pupae with punctures, however small, should be discarded.
3. Mount the pupae in a glass-bottomed dish using heptane glue.
  1. Use a 20 mL pipette tip to place a 10 mL drop of pre-prepared heptane glue (see section 1.6 above) in a line on the glass-bottomed dish.
  2. Allow the glue to dry for 5 s before transferring the dissected pupae carefully onto the heptane glue using forceps (Figure 1E).
  3. For ease of wounding and imaging, line the pupae up in a row; approximately 5 pupae are suggested but more will be manageable with experience. Note: Use of heptane glue is recommended when using upright imaging systems but is not necessary when using an inverted system. If the glue creates optical aberrations during imaging, pupae can be placed directly on the coverglass instead – the natural adhesion between the pupal tissue and glass will be sufficient in most cases for stable imaging, as long as care is taken when moving the imaging dish between microscopes.
4. For best results, mount the pupae so that the wing is flat on the coverglass with the majority of the wing surface in direct contact with the coverglass (Figure 1F). Roll the pupae using forceps to change their position to ensure the wing is mounted correctly.
5. To prevent sample dehydration during the imaging period, add a piece of absorbent filter paper soaked in distilled water to the side of the glass-bottomed dish at the end of mounting, being careful not to disturb the pupae (Figure 1E). Cover the dish with a lid. Note: Pupae are now ready for wounding and imaging.

### 3. Laser-induced Wounding of *Drosophila *Pupal Wings

1. Transfer the glass-bottomed dish containing the mounted pupae to a wide-field microscope equipped with a tunable laser ablation system.
  1. Use a pulsed-UV air-cooled nitrogen-pumped ablation laser tuned to 435 nm – see **Table of Materials** for details^11^; the precise wavelength of the light used for illumination is selected by the user via an appropriate dye cell.
2. Using brightfield optics, adjust the microscope stage controls to locate the pupal wing of the first pupa to be wounded (Figure 1F).
3. For optimal results, use an oil immersion 40X or 63X objective lens for both the imaging and laser ablation; ensure that the immersion liquid used (oil or glycerol) is consistent between ablation and imaging systems. Adjust the microscope to focus on the plane of the pupal wing epithelium nearest the glass coverslip (*i.e.* focus on the region of the epithelium to be wounded) using the fine focus control knob.
4. Using the microscope stage adjustments, position the pupal wing so that the area to be wounded is directly aligned with the known target area of the ablation laser.
5. Use an energy density attenuator slide fitted to the microscope to manually adjust the power level of the ablation light. Note: The attenuator slider has click stops and rulings to identify the relative level of attenuation and permit the use of reproducible settings.
6. Using an external manual **trigger** control, activate the ablation laser using a single brief click of the trigger to make a wound. Check for the appearance of the transient air bubble at the ablation site since wounding will normally be accompanied it. Check if the laser-induced wounding has been successful using the appropriate fluorescent filters to visualize the pupal epithelium. Note: Take care to avoid accidental exposure to the laser beams as the beam reflections can cause severe eye or skin damage.
7. If wounding is unsuccessful, vary the focal plane (moving the microscope focus slightly above or below the current focal level) and repeat the single click of the ablation trigger. Alternatively, gradually increase the laser power using the attenuator slide until the desired wound size is achieved.
8. To vary the ablation laser pulse repetition rate, use the **Repetition rate** knob on the rear control panel (which changes the rate from less than 1 pulse/s up to 60 Hz). For optimal wounding, set the pulse repetition rate to 40 Hz.
9. To generate different sized wounds, use the energy density attenuator slide to manually adjust the power level of the ablation light.
10. Refrain from wounding all of the mounted pupae and use these non-ablated pupae as unwounded controls.
11. For consistent results, regularly realign the ablation system (using the relevant operating manual). Also, clean and refill the dye resonator cell that controls the laser output wavelength.

### 4. *In Vivo *Time-lapse Confocal Imaging

1. Quickly transfer the glass-bottomed dish to an appropriate microscope for time-lapse imaging. Note: For optimal results, use a high specification confocal or spinning disc microscope equipped with sensitive detectors that can detect both GFP and mCherry fluorophores.
2. To image the entire pupal wing, use a low magnification (*e.g.* 20X) objective lens (Figure 2A). In order to image wound repair and the accompanying inflammatory response with high spatial resolution, use the oil immersion 40X (NA 1.3) or 63X (NA 1.4) objective lenses (see representative images in Figure 2B**-D**).
3. Open the appropriate image capture software associated with the microscope.
4. Using the image capture software, turn on the appropriate lasers *e.g.* the 488 nm and 561 nm lasers to visualize GFP and mCherry fluorophores, respectively (by clicking in the relevant boxes) and adjust the laser power and gain/offset settings to give sufficient fluorescent signal whilst avoiding pixel saturation; use the lowest possible laser power (in the range 5 - 20%) to minimize photobleaching and phototoxicity.
5. To capture both the repairing epithelium and inflammatory cell recruitment, set the microscope to record a z-stack using the fine focus adjustment knobs on the control panel; for optimal results, set the software (using manual button-clicks) to record z-slices through the pupal wing (minimum every 3 mm), from the top of the wounded epithelium through to the extracellular space beneath (containing migrating hemocytes) to achieve a large z-stack (in the range of 50 - 100 mm).
6. For time-lapse imaging, record z-stacks at regular time intervals (minimum every 30 s) for at least 1 h post-wounding. Note: The exact time interval between z-stacks chosen represents a trade-off between capturing the rapidly-changing cell dynamics and avoiding photo-bleaching of the samples.
7. To simultaneously image multiple pupae (including non-ablated unwounded controls), use a motorized stage (attached to the microscope) and the multi-position acquisition feature available within the imaging software. Manually set the position of each individual pupa within the software using the stage position control knobs and then manually set the appropriate z-stack limits (top and bottom) for each individual pupa.
8. Visualize the time-lapse images either during image capture or later with specialist image analysis software (such as ImageJ^32^) using z-stack projections or 3-D rendering. For example, to follow the movements of individual hemocytes (as in Figure 2C**’** and **D’**), track hemocyte nuclei using the open-access ImageJ plug-ins “TrackMate” or “Manual Tracking” (methods published in 33,34).
9. Use photoconvertible probes (such as Kaede^30^) to selectively photoconvert and label a subset of epithelial or immune cells during imaging.
  1. Open appropriate modules within the imaging software to perform the photoconversion (such as the FRAP, fluorescence recovery after photo-bleaching module) and activate the 405nm laser (by clicking in the relevant software box)^35^.
  2. Select cells to be photoconverted within the FRAP software using the square, circular or freehand selection tool. Within the FRAP software, set the time-course for photoconversion (**Bleaching**) to a single iteration/frame and set the 405 nm laser at 20% laser power. Manually click **Start the experiment** to perform photoconversion.
  3. Exit the FRAP module (click **Close**) and return to the original imaging screen within the software; use the 488 nm and 561 nm lasers to image the behavior of photoconverted and non-photoconverted cells by setting up a z-stack and time-lapse recording as above. Note: Photoconverted probes remain stable for many hours after the initial photoconversion, enabling the behavior of the photoconverted cells to be followed over time (for at least 5 h). For example, inflammatory cells in the wound can be selectively photoconverted (Figure 2F) and their behavior followed as they resolve from the injury site (Figure 2G** and H**).

## Representative Results

This protocol describes the preparation of *Drosophila *pupae for live time-lapse imaging of wound repair and inflammation *in vivo*. Using this method, it should be possible to generate multiple high-resolution movies of pupal wound closure and inflammatory cell recruitment with ease and to image the pupae for long time periods (at least 5 h) post-wounding without significant photobleaching.

### A general scheme for *Drosophila *pupa preparation

Figure 1 illustrates the optimal method for preparing *Drosophila *pupae for *in vivo *imaging. * Drosophila *white 'prepupae' are collected at '0 h' after puparium formation (APF), as the crawling larvae cease motility and adopt the stereotypical pupal shape with everted breathing appendages (spiracles) visible at their anterior-most end (Figure 1A). The white 0 h APF prepupae are allowed to develop for 18 h at 25 °C, by which time the puparium has become brown (Figure 1B) and are then dissected using fine forceps and/or microscissors (Figure 1C, to remove the protective pupal case) to reveal the optically translucent pupa within (Figure 1D). Following dissection, the pupal wings will be visible on the lateral sides of the pupal thorax (outlined in blue, Figure 1C**-D**). At this stage, other pupal body parts are also easily discernable, including the eyes, legs, and abdomen (labeled in Figure 1D). The legs are also suitable for wound inflammation studies and can be wounded using the same laser method described above. Multiple 18 h APF pupae can be mounted simultaneously within the imaging dish (Figure 1E, using heptane glue and a water-soaked filter paper to minimize dehydration) with flattest portion of wing (outlined blue) in contact with the coverslip (Figure 1F); this is particularly advantageous if the imaging microscope is equipped with a motorized stage to allow several pupae to be recorded during one single imaging period. Any pupae that have been damaged during dissection or mounting should be discarded (*e.g.* the pupa located third in the sequence, arrow in Figure 1E). Other body parts (*e.g.* eyes and legs, outlined pink) may also contact the coverslip and are available for wounding and subsequent imaging (Figure 1**F**). For experiments studying single wounds, laser-induced damages are generally best inflicted centrally in the wing (asterisk, Figure 1F), although other locations may be used if multiple wounds are to be studied.

### Sterile wounding activates a robust inflammatory response in the *Drosophila *pupal wing

In order to follow wound repair and the accompanying inflammatory response *in vivo, *the wounded wings of 18h APF *Drosophila *pupae were imaged using confocal time-lapse microscopy (Figure 2A**-H**). At this pupal stage, the wing epithelia are simple flat sheets of cells (labeled here using GFP-tagged E-cadherin to mark individual cell boundaries, wing margin outlined in Figure 2A). Even in unwounded wings (low magnification, **Figures 2A and 2A'**), large numbers of migratory innate immune cells (hemocytes, labeled using *srp-Gal4 *driven cytoplasmic GFP, green, and nuclear RFP, magenta) are found within the hemolymph (insect blood) occupying the intervening extracellular space (Figure 2A and** 2A'**). Laser-induced injury to the pupal wing epithelium (wound margin outlined in white, Figure 2B**-D**) stimulates a rapid migration of hemocytes to the wound site (Figure 2B**-D** and immune cell nuclei in Figure 2B**'-D'**). Labeling the hemocytes with both a nuclear marker (*e.g.* the nuclear RFP 'red-stinger') in combination with a cytoplasmic or cytoskeletal marker (*e.g.* cytoplasmic GFP or GFP-tagged Moesin) is particularly advantageous as it allows the simultaneous tracking of hemocyte nuclei (for automated analysis of hemocyte behavior *e.g.* migration speed and directionality) and the visualization of hemocyte morphology. The latter is important as it allows us to determine whether hemocytes are phagocytosing necrotic debris (Figure 2C, inset) at the wound edge (or microbes in the case of infection)^12^,^23^ and also allows us to follow their protrusive behavior as they extend fine filopodia or lamellipodia at their leading edge as they migrate towards or away from the wound.

Simple tracking of hemocyte nuclear trajectories using appropriate Image Analysis software^32^ (**Table of Materials**) demonstrates the complex spatio-temporal dynamics of the inflammatory response, similar to that reported previously for wounded embryos^9^,^36^ (multi-colored tracks, Figure 2C' and **D'**). Within just 30 min of wounding, hemocytes located closest to the injury site begin directed migration towards the wound (Figure 2C'). However, with increasing time post-injury, hemocytes located progressively further away from the injury site also begin directed migration towards the wound (Figure 2D'). In this way, a 'wave' of immune cell responsiveness that spreads outwards from the wound edge is observed, which we envision to reflect the diffusion of the wound chemoattractant away from the injury site. These spatio-temporal immune cell dynamics provided a useful starting point for our recent study that employed sophisticated mathematical modeling ('Bayesian inference') analysis to infer novel properties of the wound attractant signal (detailed methods published in^23^). By calibrating our computational models (that linked the wound attractant gradient to hemocyte bias) to fit the observed *in vivo* immune trajectories, one could extract detailed characteristics of the wound attractant from our hemocyte trajectory data (such as the signal diffusion rate, the source, and the duration of signal production)^23^.

Moreover, by driving expression of the photoconvertible fluorophore Kaede within the immune cell lineage using *srp-Gal4 *(green, Figure 2E) we have also been able to selectively label a subpopulation of these migratory wing hemocytes (such as those recruited to the wound site; E, before UV-induced photoconversion and F, post-photoconversion). We can then follow the behavior of these hemocytes over time (magenta, Figure 2G**-H**) as they resolve away from the wound site (arrows, Figure 2G and **H**) and compare how their behavior is different from those non-photoconverted cells that don't reach the injury site. We have used this *in vivo *labeling technique to demonstrate that hemocytes recruited to an initial wound are temporarily desensitized to a second wound generated 90 minutes later^23^, although this differential labeling technique could also have many other insightful applications in the future (see Discussion).

Using this approach, we can also simultaneously follow the spatio-temporal dynamics of wound repair or 're-epithelialisation' (Figure 2B**-D**). Here ubiquitously expressed GFP-tagged E-cadherin labels the cellular adherens junctions throughout the wing epithelium and allows the shapes of individual epithelial cells to be followed over time. The wound edge is easily identified as the junction between intact GFP-labeled epithelial cells and unlabeled debris (white dashed line, Figure 2B**-D**). For the majority of wounds, the wound begins to re-epithelialize within 1 h of injury and the wound edge advances inwards (Figure 2D); wounds of this size will typically heal within 2 - 3 h of injury^23^. Wound closure in both embryos and pupae is driven by an actomyosin contractile cable together with leading edge actin-rich filopodia^23^,^37^; these cytoskeletal dynamics can be directly visualized during wound repair using appropriate *in vivo *reporters, such as GFP-tagged Spaghetti squash (Myosin regulatory light chain) or GFP-tagged actin-binding Moesin^23^. For some particularly large wounds, however, the actomyosin cable and leading edge filopodia do not persist - these wounds become 'chronic' and never completely re-epithelialize^23^ even after much longer periods (over 24 h) of *in vivo *imaging. Interestingly, the inflammatory response associated with these non-healing wounds is markedly different from that associated with normal acute wounds^23^ suggesting that abnormal immune cell behavior could be a useful prognostic marker in the clinic. In the future, further *in vivo *analysis of chronic wounds using long-term imaging (over 24 h) in the pupal model might provide important mechanistic insight into this debilitating condition.

**Figure:**
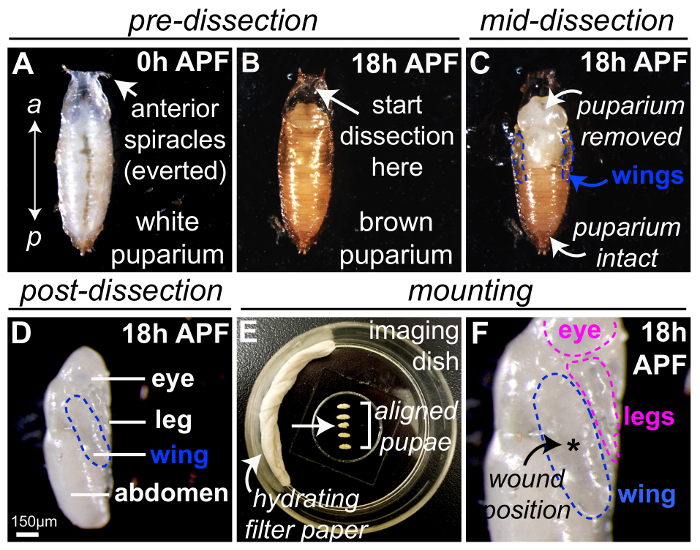

**Figure 1: *Drosophila *pupa preparation for wounding and live-imaging. **(**A**)* Drosophila *white prepupae collected at 0 h APF, with anterior end indicated by everted breathing appendages (spiracles). (**B**) After raising white 0h APF prepupae for 18 h at 25 °C, the puparium appears brown. Dissection of the pupal case should begin at the anterior-most region (arrow), indicated by the everted spiracles as the pupa proper is absent from this region, and fine forceps and/or microscissors used to remove the protective pupal case (**C**). The pupal wings will be visible on the lateral sides of the pupal thorax (blue outlines). (**D**) Pupal case completely removed from 18h APF pupa, here the pupa has been rolled 90° to show lateral side, with the wing to be imaged outlined in blue. (**E-F**) Five 18h APF pupae mounted on glass coverslip within imaging dish using heptane glue, with water-soaked filter paper to minimize dehydration (**E**). Pupa located third in the sequence (arrow) should be discarded as damage occurred during preparation. Pupae are mounted with the flattest portion of the wing (outlined blue) in contact with the coverslip (**F**) and laser-induced wounds are generated centrally in the wing (asterisk, **F**), although other locations may be used if multiple wounds are to be studied. The image in (**D**) adapted with permission from Weavers *et al.,* 2016^23^. Please click here to view a larger version of this figure.

**Figure:**
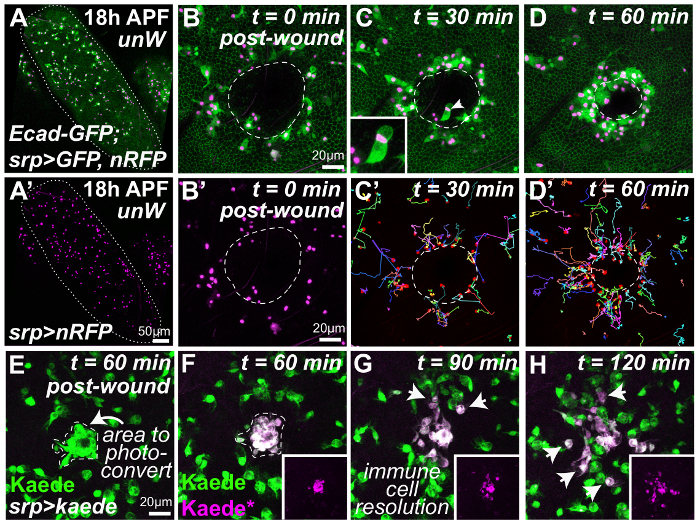

**Figure 2: Dynamic *in vivo *analysis of the inflammatory response to tissue damage.** Pupae dissected from protective pupal cases and mounted on glass coverslip (**A**) are wounded and subsequently imaged using confocal time-lapse microscopy (**B-H**). Low magnification view of the unwounded pupal wing (**A**, wing margin outlined in white) that contains large numbers of migratory hemocytes (**A**'). Laser-induced injury to the pupal wing epithelium (**B-D, **cell boundaries labeled using GFP-tagged *Drosophila *E-cadherin; wound margin outlined in white) activates a rapid inflammatory response with the migration of multiple hemocytes (*srp-Gal4 *driven expression of nuclear RFP, magenta and cytoplasmic GFP, green) towards the wound site (**B-D**; representative frames from a time-lapse movie in which each frame is a projection of 25 slices 3 μm each). Manual tracking of hemocyte trajectories (multi-colored tracks, **C'** and **D'**) indicate the complex spatio-temporal dynamics of the inflammatory response, similar to that reported for wounded embryos. Hemocytes also phagocytose necrotic cellular debris at the wound site (arrowhead, **C** and also inset). Expression of the photoconvertible fluorophore Kaede in the immune cell lineage (green, **E**, using *srp-Gal4*) enables wound-recruited hemocytes (arrow, **E**) to be differentially labeled (magenta, **F**) and followed over time as they resolve from the injury site (arrows, **G** and **H**). The following pupal genotypes were utilized: (**A-D**) *w^1118^*;*ubi-DE-cad-GFP, srp-Gal4>UAS-GFP(II); UAS-nRFP(III)* and (**E-H**) *w^1118^;srp-Gal4(II); UAS-Kaede(III). *Images adapted with permission from Weavers *et al.,* 2016^23^. Please click here to view a larger version of this figure.

## Discussion

The acute inflammatory response to tissue damage is a complex and highly dynamic process that is essential to orchestrate the repair of injured tissues, including the clearance of necrotic debris and the fight against infection. To fully understand and unravel fundamental aspects of this response, it is crucial that studies are performed *in vivo *on 3-dimensional living samples in order for the precise behavior and interactions of the various cell lineages involved to be followed accurately over time. Real-time analysis of these cell dynamics allows a more detailed characterization of mutant phenotypes than static single time-points from fixed samples using classic immunohistochemistry techniques. Traditionally, most live-imaging studies using the genetically tractable *Drosophila *model have used the embryonic stage of fruitfly development due to its optical translucency and immobility compared to the later developmental stages^4^,^5^. However, more recently our group and others have developed the *Drosophila *pupa as a novel model to perform high resolution and long-term imaging of wound repair and inflammation simultaneously *in vivo*^8^,^22^,^23^. This emerging approach offers an exciting long-term potential for unraveling fundamental aspects of the inflammatory cell behavior and can be further adapted to investigate the dynamic behavior of other cell lineages (such as *Drosophila *adipocytes^38^) following tissue injury.

There are a number of critical factors during the preparation and imaging of wounded *Drosophila *pupae that will determine the quality of the imaging outcomes described above. Arguably the most difficult step of the described protocol is the careful dissection and precise positioning of the pupae prior to wounding and imaging. Pupae at this developmental stage are extremely fragile and even minor accidental damage to the pupae during preparation stages will significantly impair the experiment; any pupae that may have sustained unintentional damage must be discarded from the experiment since this damage could activate its own inflammatory response, which might lead to more wide-spread (or even systemic) effects on inflammatory cell behavior elsewhere in the pupa. Due to the ongoing development of the pupae utilized in these experiments (which are undergoing significant tissue rearrangements to prepare tissues for adulthood), occasionally pupae will move during the course of imaging. Pupal rolling is, however, more likely to occur if pupae have not been mounted correctly with the flattest surface of the wing (or other tissue to be imaged) in the direct contact with the coverglass; use of heptane glue to stabilize the pupae on the cover glass should minimize this undesirable movement. For this reason, great care must also be taken to avoid dislodging the pupae from their carefully aligned positions when moving the samples between microscopes; ideally, the wounding laser will be attached to the same microscope to be used for subsequent time-lapse imaging and photo-conversion.

In addition to the proficiency of the pupal dissection and mounting steps, the exact genotype of the *Drosophila *pupae used will have a significant effect on the quality of the imaging data generated. For example, the number of copies of the *Gal4* driver and UAS constructs (*e.g.* UAS-GFP or UAS-Kaede) within an individual pupal genotype will determine the signal-to-noise ratio during subsequent imaging. As a general rule, the more copies of a *Gal4* or *UAS* construct present, the greater the total level of fluorescent protein (*e.g.* GFP or Kaede) within the tissue. The optimal level of fluorescent protein will, however, be a careful balance between elevating tissue fluorescence sufficiently to allow high quality imaging (enabling the use of lower laser powers, reduction in photobleaching and imaging over extended time periods) but without causing fluorophore-induced cellular toxicity; the optimal number of Gal4 and UAS constructs in each experiment will vary according to the particular drivers and fluorophores being used. Care should be taken to raise the pupae at 25 °C (or above, up to 29 °C) because the Gal4-UAS system is temperature sensitive and will be ineffective at lower temperatures^31^. In order to achieve additional levels of control over the tissue or time-specificity of *Gal4-*driven expression, the Gal4-UAS system repressor Gal80 can also be included within the pupal genotype^39^. Gal80 can either be used to repress Gal4 activity within a particular tissue (using a tissue-specific Gal80) or at a particular time (using a temperature sensitive Gal80). The Gal4-UAS system can be further combined with other independent binary systems (such as the LexA-*lexAop* and QF-*QUAS* systems) to generate *Drosophila *which has multiple constructs (*e.g.* fluorophores, RNAi lines or other genetic constructs) expressed simultaneously in a range of different tissues^39^.

Use of this new *Drosophila *pupal model offers a number of advantages over the more traditional embryo approach. Compared to the short-term imaging (up to 3 h) available in stage 15 embryos (the stage at which most embryonic wounding studies are performed), pupae can be imaged over significantly longer periods of time (in principal until adulthood after 96 h of pupal development). Moreover, far greater numbers of hemocytes (*Drosophila *innate immune cells) are present within pupal tissues (and available for imaging) compared to the more limited number present within the embryo and this has allowed us to gather significantly more imaging data on hemocyte behavior using the same total number of specimens. Crucially, this, in turn, has enabled us to apply more sophisticated mathematical modeling to analyze hemocytes behavior and extract novel features of the wound attractants and inflammatory response that would have otherwise remained experimentally inaccessible^23^. Another advantage of the pupal model is that RNAi-mediated gene knockdown is considerably more efficient than at earlier embryonic stages, allowing improved analysis of tissue or time-specific gene inactivation using binary systems such as the Gal4-UAS system^39^. The efficiency of RNAi at this stage thus opens up the potential to perform large scale (or even unbiased genome-wide) RNAi screens to search for novel players involved in either wound repair or inflammatory cell behavior.

However, *Drosophila *pupae clearly cannot be used to study the phenotypes resulting from genetic mutations which are embryonic lethal; functional and live-imaging studies of genes that are essential for embryonic development must therefore still be performed in embryos, unless RNAi-mediated gene knockdown in a time or tissue-specific manner permits development to occur through to pupal stages. The embryo also remains the model of choice to study and live-image certain features of immune cell behavior, including the developmental dispersal of immune cells from their origin, contact-inhibition of locomotion and phagocytosis of apoptotic corpses generated during developmental tissue sculpting^5^,^8^ which have not yet been observed in pupal models. Although studies in *Drosophila *larvae and adults have provided an important insight into the mechanisms underlying wound repair and inflammation^40^,^41^,^42^,^43^,^44^ live-imaging studies at these stages have proven difficult due to the inherently mobile nature of the samples. Whilst larvae can be anesthetized to allow brief periods of live-imaging, due to the temporary nature of the anesthesia, only short snapshots of the live wound repair or inflammatory response can be visualized^45^. A recent study has now developed an improved protocol that permits longer-term imaging of larval wound healing^46^, although preparation and imaging still remain considerably more challenging than in embryos or pupae. In the long-term, we envision that by utilizing the most appropriate developmental stage to address each specific question, studies in all four of these different *Drosophila *stages - from embryos through larval and pupal periods to adulthood (each with their own unique advantages and limitations) - will provide complementary insights into the molecular and cellular mechanism driving wound repair and inflammation.

In future, this protocol for wounding and long-term imaging of *Drosophila *pupae can be easily adapted to study a range of inflammation-related phenomena and has far-reaching potential for uncovering novel features of the inflammatory wound response. The combination of long-term imaging, together with the application of photoconvertible fluorophores (such as Kaede), are of great value for understanding the dynamics of innate immune cell behavior and in particular, the far less understood resolution phase of the wound inflammatory response. By specifically labeling either individual or subpopulations of immune cells (such as those recruited to a wound) it will be possible to analyze how exposure to one environmental cue (such as a corpse or injury) affects the immune cell's subsequent response to a later cue. The inflammatory behavior of *Drosophila *hemocytes can be altered by previous experiences - for example, they are primed to respond to tissue injury by prior phagocytosis of apoptotic corpses during development^12^ but it remains to be seen whether other environmental cues induce similar priming events. Although studies of pupal wounds so far have focused on the innate inflammatory response, the pupal wing model also provides an ideal opportunity to both live-image and dissect the mechanisms underlying epithelial wound repair. Moreover, this pupal imaging method could also be adapted to investigate the dynamic behavior of other cell lineages in response to tissue damage^38^, either in the pupal wing itself or other easily accessible pupal tissues (such as the eyes, legs or thorax). Finally, by combining the genetic tractability of *Drosophila *together with the ease of long-term pupal imaging, novel epithelial repair or inflammatory regulators could be uncovered through the application of unbiased genome-wide knock-down approaches.

## Disclosures

The authors declare they have no competing conflicts of interests.
